# Immuno-Pathogenesis of Chronic Inflammatory Skin Diseases: Novel Molecular Targets and Biomarkers

**DOI:** 10.3390/life12060891

**Published:** 2022-06-15

**Authors:** Annunziata Raimondo, Serena Lembo

**Affiliations:** Department of Medicine, Surgery and Dentistry, “Scuola Medica Salernitana”, University of Salerno, 84081 Baronissi, SA, Italy; slembo@unisa.it

Chronic inflammation has a crucial pathogenetic role in many diseases, including cutaneous chronic inflammatory disorders, such as psoriasis, atopic dermatitis, hidradenitis suppurativa, and chronic urticaria. Their incidence, as well as their comorbidities, is constantly growing. The pathogenesis of these diseases is very complex, and many aspects are still poorly understood. They are characterized by an interaction among genetic, immunological, and environmental aspects. In addition to genetic predisposition, development, and maintenance are associated with immune dysregulation and hypersensitivity, skin barrier defects, and environmental triggers [1]. Their chronically relapsing nature and the unsatisfactory treatment experiences cause frustration and disappointment in patients and their family members, which in turn may negatively influence the course of the disease. For all these reasons, a multidisciplinary long-term and persistent management is necessary. Increasing information on the pathophysiology and the expansion of biotechnology and pharmacology have collectively contributed to the development of new pharmacological agents: biologics targeting cytokines and small molecules have been already approved, and multiple clinical trials are in progress [2]. However, a cross-sectional, real-world survey on patients with moderate to severe atopic dermatitis revealed that more than 50% had uncontrolled disease: the need for more efficacious and safe treatments is evident [3]. The deeper investigation of the complex pathogenetic mechanisms allows the identification of new therapeutic targets, as well as of novel biomarkers with diagnostic significance, predictive value of disease severity, and of response to the therapy. The aim of this Special Issue is to collect interesting research studies focusing on the identification of novel biomarkers in inflammatory skin diseases with diagnostic and/or predictive significance to improve clinical practice and to support evidence-based treatments. Ćesić et al. performed a case–control study aimed to compare differences in salivary microbiota between chronic spontaneous urticaria (CSU) patients and healthy controls (HC) [4]. Twenty-three participants (13 patients with CSU and 10 HC) were enrolled. Salivary microbiota was determined by the molecular approach targeting 16S ribosomal RNA, and terminal restriction fragment length polymorphism (T-RFLP) analysis. Alpha diversity of salivary microbiota in CSU patients was significantly reduced compared to HC, resulting in alteration of the community composition. Species richness determined via the Shannon index was significantly reduced in the CSU group; the dominant phylum in the CSU group was Proteobacteria, in contrast to the oral microbial composition of healthy individuals, where the most abundant microbes at the phylum level are Firmicutes. This study demonstrated how dysbiosis of salivary microbiota may contribute to a dysregulated immune system in CSU. Valenzuela et al. presented a cross-sectional study comparing the gingival crevicular fluid (GCF) levels of IL-18, soluble (s)ICAM-1, and sE-selectin in psoriatic patients (*n* = 42) and healthy controls (*n* = 39) [5]. Psoriatic patients presented higher concentrations of IL-18 and lower concentrations of sE-selectin compared to controls (*p* < 0.05). No differences were found in the levels of sICAM-1 between the two groups (*p* > 0.05). Psoriasis was associated with IL-18 and E-selectin levels regardless of periodontal status, age, and smoking habit (*p* < 0.05). The areas under the receiver operating characteristic curve (ROC) for IL-18 and sE-selectin were 0.77 and 0.68, respectively. The authors suggested IL-18 and sE-selectin levels in the GCF as promising biomarkers for psoriasis. In another interesting research published in this SI, Bauer et al. described the expression and secretion of TSLP during EGFR inhibition and presented an ex vivo model, which mimics the early events after barrier insult [6]. Skin explants floated on culture medium at 32 °C released TSLP in parallel to the activation of the resident Langerhans cell network. Moreover, they showed the up-regulation and activation of the AP-1 family of transcription factors during atopic-like skin inflammation and its involvement in TSLP production from the skin explant cultures. Inhibition of the c-Jun N-terminal kinase pathway led to a dose-dependent blunting of TSLP release. These data indicate the involvement of AP-1 during the early stages of atopic-like skin inflammation and highlight a novel therapeutic approach by targeting it. Therefore, skin explant cultures mimic the early events during skin barrier immunity and provide a suitable model to test therapeutic intervention. Moreover, this SI includes an interesting narrative review paper in which Constanza Jiménez et al. reported the available literature to identify biomarkers in GCF and saliva to diagnose psoriasis [7]. Selected research studies showed that in the saliva of healthy individuals and those with psoriasis, there were differences in the levels of inflammatory cytokines, immunoglobulin A, and antioxidant biomarkers. In GCF, individuals with psoriasis showed higher levels of S100A8, IL-18, and sE-selectin in comparison to healthy individuals, independent of periodontal status. In terms of microbiological composition, the saliva of psoriatic individuals demonstrated a differential salivary microbiota. The 16s ribosomal RNA sequencing technique and posterior linear discriminant analysis demonstrated that Actinomyces, Saccharibacteria, Streptococcus and Lactobacillus taxa are associated with healthy subjects, while Prevotella, Neisseria, and Agreggatibacter are associated with the salivary microbiota of psoriatic subjects. More studies are required to determine an adequate panel of biomarkers to use in saliva or GCF for psoriasis. All these studies highlight how the identification of peripheral markers with possible predictive value of disease severity, disease progression, risk of developing comorbidities, and response to therapy is very important. This field of research is very difficult, but fascinating at the same time. It will provide important additional knowledge that could significantly improve the clinical and therapeutic management of these diseases.
